# Vending machine backgrounds: nudging healthier beverage choices

**DOI:** 10.1007/s12144-023-04420-8

**Published:** 2023-02-28

**Authors:** Ryan Calabro, Eva Kemps, Ivanka Prichard, Marika Tiggemann

**Affiliations:** 1 Psychology, College of Education, Psychology and Social Work, School of Psychology, Flinders University, GPO Box 2100, SA 5001 Adelaide, Australia; 2 Health & Exercise Sciences, College of Nursing and Health Sciences, Flinders University, Adelaide, Australia

**Keywords:** Nudging, Vending machine, Beverage choice, Soft drink

## Abstract

Soft drink overconsumption is a growing public health concern. The present research investigated whether priming nudges could decrease soft drink choices from a vending machine. We compared the effect of six vending machine wraps (Mount Franklin ™ logo, Coca-Cola™ logo, picture of water, picture of soft drink, blue, red) on beverage choice against a black (control) computerised vending machine display. In two studies, young adult participants (17 – 25 years) were recruited from [removed for blind review] (Study 1, *n* = 142, Study 2, *n* = 232). Participants were randomly allocated to choose a beverage from one of the wrap conditions. They also rated how much the beverage was liked and how often it was consumed (Study 1), or rated the refreshing value, healthiness, taste, and energy of each beverage in the vending machine (Study 2). We predicted that wraps referencing water would produce healthier choices and those referencing soft drink would result in unhealthier choices. Contrary to these predictions, the type of vending machine wrap did not significantly influence beverage choice in Study 1. However, viewing the black vending machine wrap resulted in significantly more caffeine-based selections in Study 2. Other significant predictors of the choice of beverage were how often the beverages were consumed and how much they were liked (Study 1), as well as their perceptions of the taste, healthiness and refreshing value (Study 2). The finding that the black vending machine produced more caffeine-based beverage choices demonstrates, in principle, that color-based priming nudges could influence beverage choices.

## Introduction

Around the world, there is growing public health concern about the overconsumption of soft drinks (drinks that contain carbonated water and are high in sugar) (Tahmassebi & BaniHani, 2020). Soft drinks are primarily consumed by adolescents and young adults (Australian Bureau of Statistics, 2018). Consuming too much soft drink can lead to various health issues because of the high content of sugar, such as diabetes, tooth decay, and obesity (Basu et al., 2013; Çetinkaya & Romaniuk, 2020). In an attempt to mitigate this public health concern, the World Health Organization (WHO, 2015) made a strong recommendation that people limit their daily intake of free (added) sugar to 10% of their total energy intake, equating to roughly 50 g or 12 tsp of sugar.

Current strategies to reduce soft drink consumption include taxation, restricting access, and using front-of-package labels. Taxing soft drink has become increasingly common around the world, with evidence supporting its effectiveness in reducing soft drink consumption (Teng et al., 2019). However, soft drink taxation has been shown to shift purchasing behavior to other high sugar items (Nakhimovsky et al., 2016). In addition, although restricting access to soft drinks in schools can successfully reduce soft drink consumption, it does so only within the school environment, with consumption often shifting outside of school (Micha et al., 2018). Front-of-package labels have also been shown to effectively reduce soft drink purchases in lab based studies, with some countries now mandating nutritional warning labels on soft drinks (Temple, 2020). However, there is limited research to support the effectiveness of these warning labels in real world applications (Temple, 2020).

The aforementioned approaches tend to focus on large and policy driven changes. Other less common strategies focus on altering the environment or the presentation of soft drinks. These closely align with the principle of ‘nudging’ and are less obvious to consumers than removing the option to choose soft drinks or increasing the financial cost for those who choose them. Nudging is a behavior change strategy that attempts to alter a person’s behavior in a predictable way without removing any options or changing economic incentives (Hummel & Maedche, 2019). Nudging has shown some success in bringing about behavior change across a range of domains such as road safety, energy use and healthy food choices (Hansen & Jespersen, 2013; Hummel & Maedche, 2019).

One clear benefit of nudging is that it can encourage a sense of autonomy and thus is less likely to result in reactance; engaging in the desired behavior (e.g., healthy eating) involves little or no effort. In addition, in comparison to other behavior change strategies, nudging requires less self-regulation or self-control, which would otherwise lead to a state of fatigue known as ego-depletion (Moller et al., 2006). Furthermore, nudging does not make use of reward or punishment to encourage the desired behavior. According to Deci and Flaste (1995), pursuing a behavior for something other than reward or punishment leads to the behavior being performed more, with increased enjoyment while doing so. Moreover, recent research has made a link between self-support approaches (such as nudging) and better psychological outcomes (Behzadnia & FatahModares, 2022).

There are many different types of nudges (for a review see Wilson et al., 2016). One particular type and the focus of the present study is a priming nudge. Priming nudges are subconscious cues that can be physical, verbal, or sensory, which are designed to subtly guide a particular choice. They differ from other types of nudges because they do not change the default option, provide an incentive, enforce commitment, or establish norms. Instead, examples of priming nudges include changing the location of food items on a menu (Gynell et al., 2022), changing the shelf positions of food and beverages in shops and supermarkets, and using color to promote certain food consumption behaviors (Wilson et al., 2016).

To date, several studies have shown that priming nudges can successfully promote the consumption of healthier beverages. Two of these reported an effect of the color of plates and cups on the amount participants consumed from them (Akyol et al., 2018; Genschow et al., 2012). A third found that the existence of snack and beverage brand logos in schools was associated with an increased consumption of unhealthy foods (e.g., chocolate, and salty snacks), but not beverages (e.g., soft drink) (Minaker et al., 2011). Brown and Tammineni (2009) examined a range of factors designed to increase the number of healthy beverage purchases from vending machines in primary schools. These included reducing the availability of soft drinks and reducing the price of healthy beverages, as well as changing the vending machine wraps (the surround of the drink display) to reflect physical activity, feature school logos, or feature one of the healthier beverage choices through branding. Brown and Tammineni (2009) found that soft drink purchases decreased and healthy beverage purchases increased. However, as the study did not isolate the effect of the vending machine wrap (primes) from changes made to availability and pricing, it remains unclear which factor(s) were responsible for the healthier beverage choices.

The aim of the present research was to investigate specifically the effect of vending machine wraps on beverage choice. Vending machines are readily available across many different environments, such as shopping centres, factories, office buildings, airports, and schools (Statistic Brain Research Institute, 2017), thereby providing easy access to on-the-go snacks and drinks. People often purchase soft drinks from vending machines (Grand View Research, 2019), and may be unknowingly influenced by features incorporated into the wrap of the vending machine. Yet research into the effects of priming nudges on selections from vending machines is limited. A more general review of factors that influence vending machine choices (Hua & Ickovics, 2016) identified only one study that focused on priming nudges, namely that of Brown and Tammineni (2009) mentioned above.

In the present research, in two studies (one laboratory, one online) we manipulated the wrap on a computerised vending machine display to feature priming nudges (beverage brand logo, picture of a beverage in a glass, or the color of a brand of beverage). In each study, participants were randomly allocated to one of seven vending machine wrap conditions and asked to choose a beverage from the vending machine without considering price. Based on the previous research (Akyol et al., 2018; Brown & Tammineni, 2009; Genschow et al., 2012), we hypothesised that brand logos, pictured beverages, or associated brand color wraps could influence beverage choices. Specifically, we predicted that participants would be more likely to choose water (a healthy option) from vending machine wraps that featured the Mount Franklin™ (a popular brand of water in Australia) logo, a picture of water in a glass, or were colored blue (a visual representation of water/Mt. Franklin™ brand) (Hypothesis 1). We also predicted that participants would be more likely to select a soft drink (an unhealthy option) from vending machine wraps that featured the Coca-Cola™ logo, a picture of soft drink (Coca-Cola™) in a glass, or were colored red (a visual representation of soft drink/Coca-Cola™ brand), relative to a black colored (control) vending machine (Hypothesis 2). In addition, we measured how much participants liked the various beverages in the vending machine and how often they consumed them (Study 1), and how healthy, tasty, refreshing and energising they perceived the beverages to be (Study 2).

## Study 1 Method

### Participants

Participants were 142 undergraduate students at [removed for blind review] who took part for course credit or a $5 reimbursement. Participants were recruited via a university research participation system for a study investigating the psychology of drink choices from vending machines. Participation was limited to young adults (17 – 25 years) to capture the primary consumers of soft drinks. The sample consisted of 118 women and 24 men with a mean age of 19.77 years (*SD* = 1.92). The mean BMI of the sample was 24.27 kg/$${\mathrm{m}}^{2}$$ (*SD* = 6.20).

### Design

The experiment used a between-subjects design. Participants were randomly allocated to one of seven experimental conditions by the random allocation feature in the survey creation software Qualtrics. The seven vending machines wraps were Mount Franklin™ logo, Coca-Cola™ logo, picture of water, picture of soft drink, blue, red, or black. The dependent variable was the type of beverage chosen (water, soft drink, caffeine-based).

### Materials

#### Vending Machine Wrap Displays

**Fig. 1:**
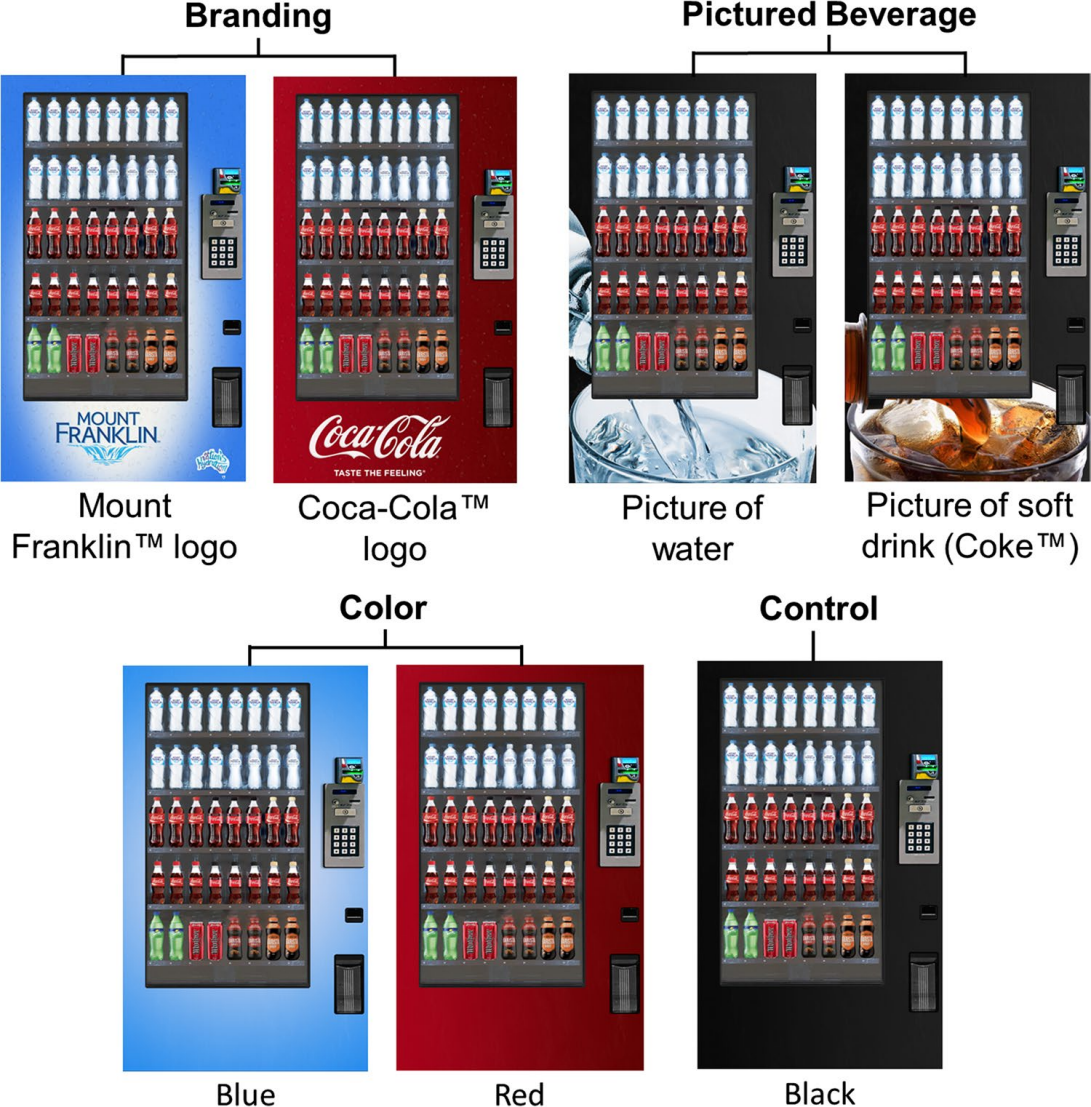
The seven vending machine conditions

Seven vending machine wraps were created: Mount Franklin™ logo, Coca-Cola™ logo, picture of water, picture of soft drink, blue, red, or black. As shown in Fig. 1, the two branding wraps featured a Mount Franklin™ or Coca-Cola™ logo and their respective slogan on a colored background that was blue for Mount Franklin™ and red for Coca-Cola™. The pictured beverage wraps featured a picture of water or soft drink (Coca-Cola™) in a glass on a black background. The color wraps were either blue or red. The control was black, i.e., the color of a vending machine without a wrap.

Each vending machine image was displayed in portrait orientation on a touchscreen computer. This allowed for a realistic representation and a larger overall image size. The beverage arrangement was identical across all vending machine conditions and was based on observations of typical vending machines in Australia. These commonly feature two rows of water, two rows of soft drink and one row of beverages that are high in caffeine. Thus, the beverage selection included water (Mount Franklin™), soft drinks (Coca-Cola™, Vanilla Coca-Cola™ and Sprite™), as well as a well-known energy drink (Mother™), and coffee beverages (Barista Bros™ Iced Coffee and Barista Bros™ Double Espresso). Because of its high caffeine content, the energy drink was categorised along with the two coffee beverages as a ‘caffeine-based’ beverage.

#### Beverage Choice Task

Participants were instructed to imagine that they were in front of a real vending machine and to choose a beverage that they would like to drink straight away, without consideration of price. Participants made their selection by touching their beverage of choice on the screen and were then asked why they chose that particular beverage. Such choice tasks in the food domain have shown high test–retest reliability (Foerde et al., 2018).

Participants were then asked to rate how often they typically consume each of the beverages in the vending machine display (rated on a 7-point Likert scale ranging from ‘never’ to ‘daily’). They were also asked to rate how much they like each of the beverages (rated on a 100 mm visual analogue scale ranging from ‘not at all’ to ‘very much’). Such ratings in the food consumption domain have demonstrated a high test–retest reliability (Foerde et al., 2018).

#### Background Information

Participants reported their age and gender, and the last time they drank anything (estimated to the nearest 15 min). They also rated how thirsty they were on a 100 mm visual analogue scale ranging from ‘not at all thirsty’ to ‘extremely thirsty’.

### Procedure

**Fig. 2:**
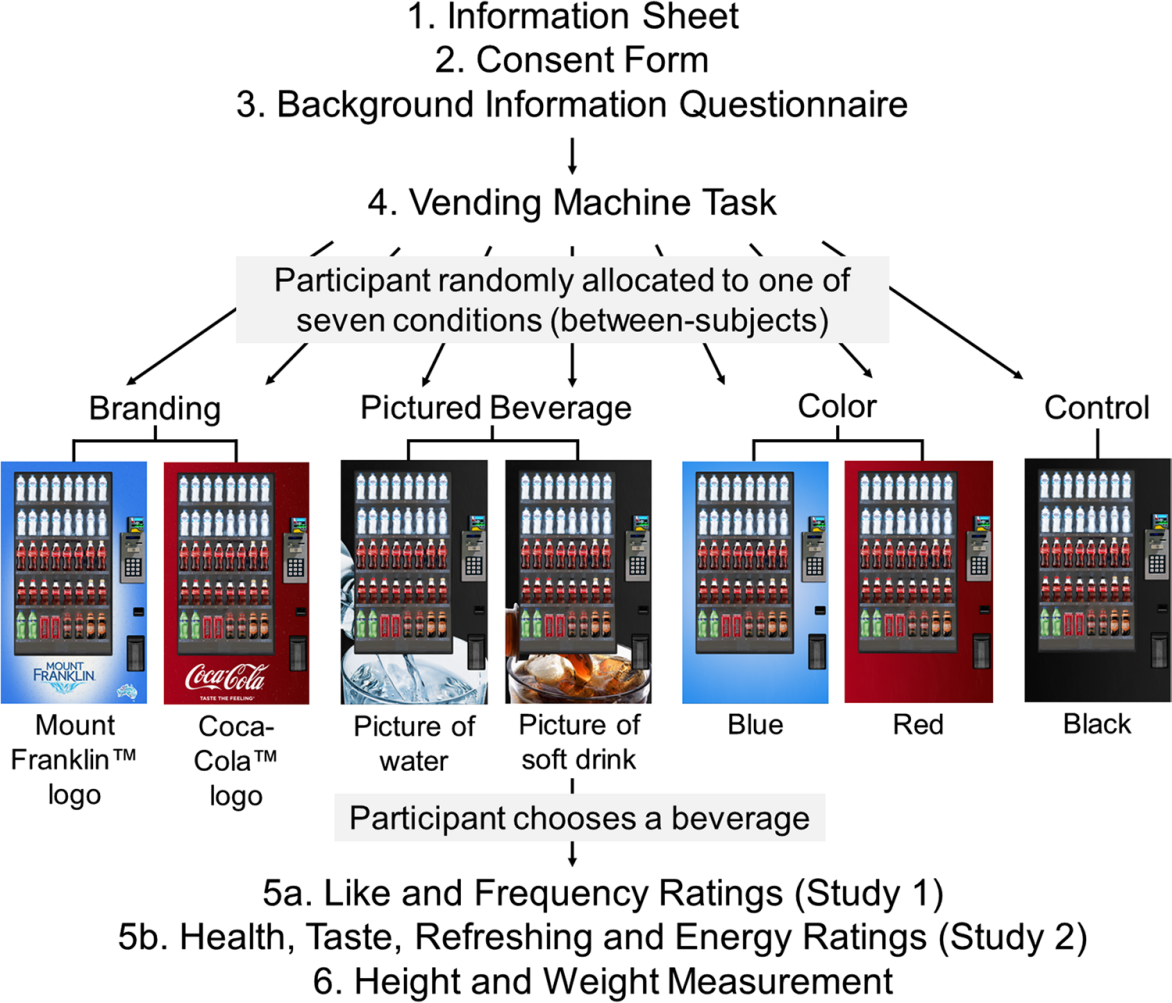
A flow chart of the procedure used in Studies 1 and 2

The study received ethics approval from [removed for blind review] University’s Social and Behavioral Research Ethics Committee (approval number 8391) and was run in accordance with the National Statement on Ethical Conduct in Human Research (2007). The procedures used in this study adhere to the tenets of the Declaration of Helsinki. Written informed consent was obtained from all individual participants included in the study. It was conducted in the Food Research Laboratory at [removed for blind review]. Figure 2 provides a flow-chart overview of the procedure. Participants were seated in front of a touchscreen computer. After reading the study information and providing written informed consent to participate in the study, participants provided some basic demographic information. They then completed the beverage choice task and post-choice questions. Finally, the participant’s height was measured by the researcher, and the participant weighed themselves and entered their weight in kg into the survey. Body mass index (BMI; kg/m^2^) was calculated using these measurements. All questionnaires were administered via Qualtrics and participants completed the study in 10–15 min.

### Data analysis

To test the effect of condition (Mount Franklin™ logo, Coca-Cola™ logo, picture of water, picture of soft drink, blue, red, or black) on beverage choice (water, soft drink, caffeine-based), a multiple logistic regression was conducted using the IBM Statistical package for Social Sciences (SPSS, version 26) software, with significance levels set at *p* < 0.05. This analysis was the most suitable due to the categorical nature of the independent and dependent variables. In addition, this type of analysis allowed us to control for potential confounds such as thirst and gender. All comparisons were conducted using the control (black) vending machine as the comparison condition, and water as the comparison beverage choice.

## Results

### Beverage choice

Overall participants mostly chose water (45.1%), followed by soft drinks (38.2%) and caffeine-based beverages (16.7%). Table 1 provides a breakdown of beverage choices per condition. The most commonly reported reason for choosing a particular beverage was because it was liked (10.4%), or because it was considered refreshing (9.7%), energising (8.3%), tasty (7.6%), or healthy (6.3%).

**Table 1:** Percentage of beverage type chosen in each condition for Study 1

Condition	Water	Soft Drink	Caffeine-based
Mt. Franklin™ logo	47.6%	47.6%	4.8%
Coca-Cola™ logo	47.6%	38.1%	14.3%
Picture of Water	47.6%	33.3%	19.0%
Picture of Soft Drink	57.1%	28.6%	14.3%
Blue	50.0%	15.0%	35.0%
Red	35.0%	55.0%	10.0%
Black	30.0%	50.0%	20.0%

### Effect of vending machine condition on beverage choice

A multinomial regression was conducted to test the effect of multiple factors (vending machine wrap condition, gender, thirst, beverage liking, frequency of consumption) on beverage choice (water, soft drink, caffeine-based). The overall model explained a significant 69.4% of the variance, *X*^2^ (28, *n* = 142) = 131.059, *p* < 0.001, and did not violate the Pearson (*X*^2^ (252, *n* = 142) = 220.795, *p* = 0.922) or Deviance (*X*^2^ (252, *n* = 142) = 158.757, *p* = 1.000) tests for goodness of fit.

The analysis showed that condition was not a significant predictor of beverage choice (see Table 2). Not surprisingly, how much water and soft drink were liked, and how often soft drink and caffeine-based beverages were typically consumed were significant predictors of beverage choice. More specifically, the parameter estimates show that participants who chose soft drink reported liking soft drink significantly more, *b* = 0.50, *p* = 0.004, *OR* = 1.052 (95% *CI*: 1.016, 1.088), and water significantly less,* b* = -0.36, *p* = 0.007, *OR* = 0.964 (95% *CI*: 0.939, 0.990), than those who chose water. The parameter estimates further show that how often a caffeine-based beverage was consumed significantly predicted a caffeine-based choice over a water choice, *b* = 2.502, *p* = 0.016, *OR* = 12.211 (95% *CI*: 1.588, 93.905).

**Table 2:** Study 1 multinomial logistic regression results to predict beverage choices (water, soft drink, caffeine-based)

Effect	Model Fitting Criteria (-2 Log Likelihood of Reduced Model)	Chi-Square	*df*	Sig
Condition	170.610	11.853	12	.458
Gender	160.839	2.082	2	.353
Thirst Rating	158.858	.102	2	.950
Liking Ratings				
Water	168.105	9.348	2	.009**
Soft Drink	168.722	9.965	2	.007**
Caffeine-based	163.910	5.153	2	.076
Frequency of Consumption				
Water	161.087	2.330	2	.312
Soft Drink	167.636	8.880	2	.012*
Caffeine-based	171.418	12.661	2	.002**

The reference category is water. *p < .05, **p < .01

## Discussion

Study 1 investigated whether priming nudges incorporated into vending machine wraps could influence beverage choice behavior. In contrast to Hypothesis 1, vending machine wraps that featured priming nudges related to healthy beverages (Mount Franklin™ logo, picture of water in a glass, or a blue colored wrap) did not result in more healthy beverage choices. Likewise, in contrast to Hypothesis 2, priming nudges related to unhealthy beverages (Coca-Cola™ logo, picture of Coca-Cola™ in a glass, or a red colored wrap) did not result in more unhealthy beverage choices. Instead, participants chose beverages that they liked and regularly consumed. The most common reasons for beverage choice were related to the refreshing, energising, taste, and health aspects of the beverage. We therefore examined these factors directly as potential drivers of beverage choice in Study 2. In contrast to the lab-based set-up of Study 1, Study 2 was conducted online to mitigate against any social desirability bias in beverage choices from the presence of the experimenter in the room.

## Study 2 method

### Participants

Participants were 232 undergraduate students at [removed for blind review] who took part for course credit or a $5 reimbursement. Participants were again recruited from [removed for blind review] the online research participation system for a study investigating the psychology of drink choices from vending machines. The sample was again limited to young adults (17 – 25 years) and consisted of 186 women and 46 men with a mean age of 19.39 years (*SD* = 2.08). Mean BMI of the sample was 24.85 kg/$${\mathrm{m}}^{2}$$ (*SD* = 6.00).

### Design, materials, procedure, and data analysis

Study 2 was conducted fully online during COVID-19 restrictions. This carried the advantages of greater anonymity and reduced possible social desirability and demand effects. The study again received ethics approval from [removed for blind review] University’s Social and Behavioral Research Ethics Committee (approval number 8391) and was run in accordance with the National Statement on Ethical Conduct in Human Research (2007). The procedures used in this study adhere to the tenets of the Declaration of Helsinki. Written informed consent was obtained from all individual participants included in the study. Design, materials, procedure, and data analysis were the same as in Study 1, except that the liking and frequency ratings were replaced by ratings of how healthy, tasty, refreshing and energising each beverage featured in the vending machine was perceived to be, based on the most common reported reasons for choosing a beverage in Study 1. These were measured using 100 mm visual analogue scales ranging from ‘not at all’ to ‘very much’.

## Results

### Beverage choice

Participants mostly chose soft drinks (42.6%), closely followed by water (41.3%), with fewer choosing caffeine-based beverages (16.2%). Table 3 provides a breakdown of beverage choices per condition. The most commonly reported reason for choosing a beverage was that it was liked (12.3%), followed by it being energising (8.5%), healthy (8.0%), thirst quenching (8.0%), and refreshing (7.2%).

**Table 3:** Percentage of beverage type chosen in each condition for Study 2

Condition	Water	Soft Drink	Caffeine-based
Mt. Franklin™ logo	34.4%	53.1%	12.5%
Coca-Cola™ logo	45.5%	36.4%	18.2%
Picture of Water	44.1%	41.2%	14.7%
Picture of Soft Drink	28.6%	51.4%	20.0%
Blue	50.0%	35.3%	14.7%
Red	51.5%	42.4%	6.1%
Black	35.3%	38.2%	26.5%

### Effect of vending machine condition on beverage choice

A multinomial regression was conducted to test the effect of multiple factors (vending machine wrap condition, gender, thirst, and the ratings of how healthy, refreshing, tasty and energising each beverage was) on beverage choice (water, soft drink, caffeine-based). The overall model explained a significant 52.4% of the variance, *X*^2^ (40, *n* = 232) = 141.355, *p* < 0.001, and did not violate the Pearson (*X*^2^ (422, *n* = 232) = 426.068, *p* = 0.436) or Deviance (*X*^2^ (422, *n* = 232) = 332.534, *p* = 1.000) tests for goodness of fit.

Overall, condition was a significant predictor (*p* = 0.046) of beverage choice (see Table 4). Specifically, the parameter estimates show that participants made significantly more caffeine-based choices than water choices in the black condition, compared to the water, *b* = -1.908, *p* = 0.044, *OR* = 0.148 (95% *CI*: 0.023, 0.952), red, *b* = -3.672, *p* = 0.001, *OR* = 0.025 (95% *CI*: 0.003, 0.229), or blue, *b* = -2.639, *p* = 0.007, *OR* = 0.071 (95% *CI*: 0.010, 0.486) conditions. Gender was also a significant predictor of beverage choice (*p* = 0.007), with the parameter estimates showing that men were more likely to choose caffeine-based beverages,* b* = 1.883, *p* = 0.003, *OR* = 6.574 (95% *CI*: 1.888, 22.890) than women. In addition, how tasty (water, soft drink, caffeine-based), refreshing (soft drink, caffeine-based), and healthy (water) beverages were significantly predicted beverage choice; energy ratings were not a significant predictor.

**Table 4:** Study 2 multinomial logistic regression results to predict beverage choices (water, soft drink, caffeine-based)

Effect	Model Fitting Criteria (-2 Log Likelihood of Reduced Model)	Chi-Square	*df*	Sig
Condition	353.846	21.312	12	.046*
Gender	342.416	9.882	2	.007**
Thirst Rating	333.843	1.308	2	.520
Health Ratings				
Water	345.759	13.225	2	.001**
Soft Drink	338.127	5.592	2	.061
Caffeine-based	333.074	.539	2	.764
Refreshing Ratings				
Water	334.464	1.930	2	.381
Soft Drink	343.929	11.394	2	.003**
Caffeine-based	346.616	14.082	2	.001**
Taste Ratings				
Water	344.123	11.589	2	.003**
Soft Drink	352.618	20.084	2	.000***
Caffeine-based	359.324	26.790	2	.000***
Energy Ratings				
Water	336.354	3.819	2	.148
Soft Drink	333.268	.734	2	.693
Caffeine-based	335.919	3.385	2	.184

The reference category is water. *p < .05, **p < .01, ***p < .001

## Discussion

In line with the findings of Study 1, the results of Study 2 did not support the hypotheses. Specifically, vending machine wraps that featured priming nudges related to healthy beverages (Mount Franklin™ logo, picture of water in a glass, or a blue colored wrap) did not result in more healthy beverage choices, and priming nudges related to unhealthy beverages (Coca-Cola™ logo, picture of Coca-Cola™ in a glass, or a red colored wrap) did not result in more unhealthy beverage choices. However, the black colored (control) vending machine did influence beverage choice, with relatively more people choosing caffeine-based beverages in this condition. We speculate that the black color of the vending machine may have influenced caffeine-related thoughts (due to caffeine being associated with the color black) and therefore potentially resulted in more caffeine-based choices. In addition, men were more likely to choose caffeine-based beverages than women. Participants also chose beverages they considered to be tasty, refreshing, and healthy.

## General discussion

The present studies aimed to examine whether priming nudges featured on a vending machine wrap could influence beverage choice behavior. The findings from Study 1 showed that vending machine wraps had no significant effect on beverage choices, while Study 2 found a significant effect for the black vending machine (control) condition only. Specifically, participants who viewed the black vending machine chose relatively more caffeine-based beverages than water. Not surprisingly, beverage choices were also driven by how much a beverage was liked, how often it was consumed (Study 1), and perceptions of its taste, refreshing qualities and healthiness (Study 2).

We had predicted that participants would be more likely to choose water (a healthy option) from vending machine wraps that featured the Mount Franklin™ (a popular brand of water in Australia) logo, a picture of water in a glass, or were colored blue (a visual representation of water/Mt. Franklin™ brand) compared to a black colored (control) vending machine (Hypothesis 1). Conversely, we had predicted that participants would be more likely to choose a soft drink (an unhealthy option) from vending machine wraps that featured the Coca-Cola™ logo, a picture of soft drink (Coca-Cola™) in a glass, or were colored red (a visual representation of soft drink/Coca-Cola™ brand), relative to a black colored (control) vending machine (Hypothesis 2). The results of neither study supported these hypotheses. Thus the present findings are inconsistent with those of Brown and Tammineni (2009). In contrast to the present studies which isolated the effect of changes in vending machine wrap, Brown and Tammineni (2009) reported on the effect of these nudges in combination with changes in beverage availability and pricing. Our findings suggest that the effects on beverage choice observed by Brown and Tammineni (2009) were most likely due to the changes in the pricing and availability of beverages rather than their vending machine wraps. Future research should test each of the above factors independently. The present findings are, however, consistent with those of Minaker et al. (2011) who found no association between the presence of beverage logos and beverage consumption in schools (although there was an association between the presence of snack logos and unhealthy food consumption). In a similar vein, Stöckli et al. (2016) showed that the presentation of a poster depicting a health-evoking nature scene, physical activity or skinny Giacometti sculptures on a vending machine resulted in healthier snack food choices. Together these findings suggest that priming nudges on vending machines may be more effective for food choices than beverage choices.

The finding that the black vending machine resulted in more caffeine choices in Study 2 supports in a general way the previously reported color effects by Genschow et al. (2012) and Akyol et al. (2018). Although we had predicted that the blue and red prime wraps would influence beverage choice positively and negatively, respectively, we found instead that the black vending machine increased caffeine-based choices. It seems likely that the black color of the vending machine may have primed or cued caffeine because it is readily associated with the color of coffee. Future research could usefully examine whether other colors could similarly influence beverage choices from vending machines; for example, a green vending machine (a color associated with healthiness) might encourage healthier beverage choices.

Interestingly, the effect of the black vending machine on caffeine-based choices was only observed in Study 2. One difference between the two studies is that participants could complete Study 1 only during working hours (9am – 5 pm), whereas they could complete Study 2 at any time of the day. Participants who completed Study 2 at night may have had a greater desire for caffeine-based beverages due to a need for energy at that time. There was indeed a large proportion of caffeine choices (> 50%) after 10 pm. As a result, participants may have been more susceptible to the black vending machine condition priming caffeine related thoughts.

Not surprisingly, participants’ beverage choices in Study 1 were largely based on how often they consumed the beverages and how much they were liked. These two factors suggest.

that beverage choices are mostly habitual, as has been shown previously for sugar sweetened beverage consumption (Dono et al., 2021; Zhen et al., 2011). In Study 2, participants’ perceptions of how healthy, tasty, and refreshing the beverages were contributed to their choices, which fits with previous research that has linked unhealthy beverage consumption behavior to perceptions of the taste of beverage (Block et al., 2013; Dono et al., 2020). Looking at the patterns of perceptions in more detail, not surprisingly water choices were the only beverage choice associated with healthiness. In contrast, soft drinks and caffeine-based beverage choices were primarily associated with how refreshing and tasty they are. This difference likely reflects the focus of marketing strategies that promote soft drinks and caffeine-based beverages as tasty and refreshing. It is therefore possible that bottled water could be made a more attractive choice if its refreshing nature was more strongly empathized. More generally, attempts to shift individuals towards healthier beverages might usefully target and increase people’s perceptions of how tasty, refreshing, and healthy beverages are.

At a practical level, the priming nudges incorporated into the vending machine wraps in the present studies may not have been sufficiently strong to override existing habitual consumption behaviors. However, the finding that a black vending machine produced more caffeine-based beverage choices demonstrates, in principle, that color-based priming nudges might be a useful tool. In the present case, if one wanted to reduce the choices of caffeine-based beverages, one could avoid the color black on vending machines, a suggestion that could be tested in a field study. If effective, this type of priming nudge may then extend to other environments such as restaurant menus, supermarkets shelves, and drink fridges in cafes. Any such change (e.g., changing the color of a display) would be easily implementable, and does not depend on government regulation or policy change. Future research might specifically examine other colors in more powerful designs. In addition, although the focus of the present studies was on soft drink choices, it should be noted that the caffeine-based beverages in the vending machine displays also contained a high amount of sugar (in addition to caffeine), similar to that of soft drink (more than 40 g of sugar per serve). Therefore, these caffeine-based beverages share the same negative health consequences as soft drinks, in addition to the potential cardiovascular risks associated with overconsumption of caffeine (Poole et al., 2017).

The present research has some limitations. First, the samples consisted of predominantly female students of mostly average weight. Hence, the priming nudges (e.g., logos, pictured beverages, and color) may differentially affect beverage choices of children and older adults. Thus, future research should investigate whether the current findings generalise to other demographic groups. Second, the limited range of beverages offered in the vending machine displays may have restricted participant choice but was modelled on actual vending machines. In addition, some brands of beverage were exclusive to the Australian market. Third, participants in the present studies never had the opportunity to consume their chosen drink. Nevertheless, hypothetical choices have been shown to activate similar brain systems as real choices (Kang et al., 2011), and thus are a valid predictor of actual choices. Fourth, the present studies were conducted in the lab or online. Future research should seek to test the effects of vending machine wraps on actual machines in the field. Finally, priming nudges may influence motivational regulations that contribute to healthy beverage choice behavior. In particular, future research could adopt a Self-Determination Theory (Deci & Ryan, 1985) approach to investigate whether priming nudges affect beverage choices through autonomous or controlled motivation.

In conclusion, the present studies offer little evidence that vending machine wraps can shift beverage choices. However, they might do under certain circumstances. Specifically, the black vending machine wrap resulted in more caffeine-based choices than water and thus should be avoided. This is a promising start for priming nudges as a pathway towards promoting healthier beverage consumption behavior.

## Data Availability

The data analysed during the current study are available from the corresponding author on reasonable request.
